# The impact of low plasma atherosclerosis index on hemorrhagic transformation after endovascular treatment of large artery atherosclerotic stroke

**DOI:** 10.3389/fneur.2025.1694640

**Published:** 2025-11-06

**Authors:** Jianqiang Hu, Mingqing Cheng, Shuyu Ma, Jiawei Zhang, Xin Miao, Tingting Liu, Kefangyuan Zheng, Yalan Fang, Jin Zhang

**Affiliations:** 1 Clinical College, Shanxi Medical University, Taiyuan, China; 2 Department of Neurology, First Hospital of Shanxi Medical University, Taiyuan, China; 3 Department of Neurology, Second Hospital of Shanxi Medical University, Taiyuan, China

**Keywords:** the atherogenic index of plasma, endovascular treatment, hemorrhagic transformation, infarct volume, large-artery atherosclerotic stroke

## Abstract

**Objective:**

The plasma Atherosclerosis Index (AIP) reflects lipid metabolism, but its relationship with hemorrhagic transformation (HT) after endovascular treatment (EVT) in large artery atherosclerotic stroke (LAA) is unclear. This study aims to assess AIP’s potential in predicting HT occurrence.

**Methods:**

This retrospective study analyzed 467 LAA patients. The patients were grouped based on infarct volume (small, moderate, and large). Univariate and multivariate logistic regression models evaluated the relationship between AIP levels and HT risk. Additionally, we analyzed hemorrhagic infarction (HI), parenchymal hemorrhage (PH) and symptomatic hemorrhagic transformation (sHT) to evaluate the relationship between AIP and the risk of them.

**Results:**

Among the 467 patients, 199 experienced HT after EVT. After adjusting for confounders in the multivariate logistic regression model, lower AIP (OR = 0.20, *p* = 0.001) was significantly associated with an increased risk of HT. Subgroup analysis revealed that in the small infarct volume group, lower AIP (OR = 0.11, *p* = 0.012) was significantly associated with the risk of HT. AIP was also significantly associated with the risk of HI (OR = 0.12, *p* = 0.003) and sHT (OR = 0.11, *p* = 0.002) in HT.

**Conclusion:**

Lower AIP levels are an independent risk factor for HT after EVT in LAA patients, especially in the small infarct volume group. Moreover, AIP is associated with HI and sHT.

## Introduction

Large artery atherosclerotic stroke (LAA) arises from the formation of atherosclerotic plaques within significant arteries, resulting in vessel narrowing or occlusion and subsequently leading to brain ischemia (1, 2). This stroke subtype is prevalent among ischemic strokes and is characterized by high rates of recurrence and long-term disability. In recent years, endovascular treatment (EVT) has emerged as an essential and effective strategy for improving reperfusion success and enhancing patient outcomes (3). Despite its benefits, hemorrhagic transformation (HT) following EVT remains one of the most serious and frequent complications, potentially compromising clinical results and hindering functional recovery (4).

Previous studies have reported that the occurrence of HT is related to various factors, including age, blood pressure, baseline NIHSS score, large infarct size, thrombolysis treatment, and intracranial blood flow velocity (5–7). However, the relationship between blood lipids and HT remains controversial. Some studies suggest that low levels of triglycerides (TG) and low-density lipoprotein cholesterol (LDL-C) may increase the risk of HT after cerebral infarction (8, 9), while others have failed to find significant associations (10). In recent years, increasing attention has been focused on the Atherogenic Index of Plasma (AIP), a biomarker reflecting lipid metabolism and predicting atherosclerosis risk. The formula for calculating AIP is log (TG/HDL-C) (11–13). Some studies have suggested that AIP is associated with the incidence of cerebrovascular events and clinical outcomes in large artery atherosclerotic stroke, and it may be superior to traditional lipid parameters (14–16). However, there is limited research on the correlation between AIP and HT after EVT in large-artery atherosclerotic stroke patients.

Therefore, this study aims to investigate whether a lower AIP level increases the risk of HT after EVT in large artery atherosclerotic stroke patients and to explore the effects of AIP through subgroup analyses under different conditions.

## Method

### Study population

This study was approved by the Ethics Committee of the First Hospital of Shanxi Medical University. A retrospective collection of patients treated with EVT at the First Hospital of Shanxi Medical University between January 2020 and December 2024 was conducted. The ischemic stroke patients were classified according to the TOAST classification, and their classification was primarily confirmed through medical records. The inclusion criteria were: (1) EVT treatment within 24 h; (2) age ≥18 years; (3) confirmed large artery occlusion stroke via computed tomography angiography (CTA), magnetic resonance angiography (MRA), or digital subtraction angiography (DSA). The exclusion criteria were: (1) posterior circulation stroke; (2) cardioembolic, undetermined, or other types of stroke; (3) lack of head CT or MRI examination after surgery; (4) missing lipid profile data; (5) history of intracranial hemorrhage.

### Data collection

Demographic characteristics include age, sex, and BMI. Clinical features include smoking history, admission blood pressure, preoperative NIHSS and ASPECTS scores, antiplatelet and lipid-lowering therapy prior to onset, and intravenous thrombolysis implementation. Medical history includes hypertension, diabetes, history of stroke, and coronary heart disease. Laboratory data include TG, total cholesterol (TC), LDL-C, high-density lipoprotein cholesterol (HDL-C), homocysteine, and albumin levels. Surgical characteristics include onset-to-puncture time (OPT), surgery duration (time from entering the operating room to completion of puncture), and the number of endovascular treatments. Imaging characteristics include HT and infarct volume. All blood samples were collected within 24 h after hospital admission while fasting, and were analyzed by laboratory specialists. Surgical data were extracted from the surgical records. The number of endovascular treatments included thrombectomy, stent retrieval, balloon angioplasty, stent implantation, and intra-arterial thrombolysis. Symptomatic hemorrhagic transformation (sHT) is defined as HT after surgery accompanied by worsening neurological deficits and increasing in the NIHSS score (≥ 4 points).

### Imaging data

Brain imaging data for all patients were obtained via CT scanning. All patients underwent head CT within 24 to 48 h after EVT, with a follow-up CT conducted 5–7 days post-surgery to differentiate between HT and contrast agent staining. HT is defined by the appearance of high-density shadows in low attenuation areas on follow-up CT scans. The diagnosis of HT is based on the European Acute Stroke Study I (ECASS II) criteria (17). HT is classified into two main categories: Hemorrhagic Infarction (HI) and Parenchymal Hematoma (PH). HI is further subdivided into HI1, which refers to small, punctate hemorrhages at the infarct border, and HI2, which involves confluent punctate hemorrhages within the infarct area without significant mass effect. PH is divided into PH1, where the hematoma occupies less than 30% of the infarcted area with mild mass effect, and PH2, where the hematoma exceeds 30% of the infarct area and is associated with significant mass effect.

Infarct volume was calculated using the open-source software 3D-Slicer (https://download.slicer.org/). The infarct area for each slice was manually segmented, and the area was measured for each slice. The infarct volume for each layer was calculated by multiplying the infarct area by the slice thickness. The total infarct volume was obtained by summing the volumes from all slices (unit: mL). CT images from the 5–7 day postoperative head CT scan, which clearly defined the infarct area, were prioritized to ensure segmentation accuracy. When HT occurred, the total infarct volume was first calculated, followed by the hemorrhagic region volume. The non-hemorrhagic region volume was then determined by subtracting the hemorrhagic region volume from the total infarct volume.

### Statistical analysis

In this study, continuous variables are presented as mean ± standard deviation (SD) or median (interquartile range, IQR), and categorical variables are presented as number (*n*) and percentage (%). The differences between groups were assessed using Student’s *t*-test (for continuous variables), Mann–Whitney U test (for continuous variables), and Chi-square test (for categorical variables). The AIP (Atherogenic Index of Plasma) was calculated using the formula: log (TG/HDL-C). Single-variable and multivariable logistic regression analyses were conducted with HT as the dependent variable. Significant variables from univariate analysis were included in the multivariate logistic regression model to calculate the odds ratio (OR) and 95% confidence intervals (CI) for the association between AIP and HT. In subgroup analysis, we evaluated the difference in HT risk between the three AIP groups (low, medium, and high) across different infarct volume subgroups. The infarct volume was divided into three subgroups: small infarct volume (0–15 mL), medium infarct volume (15.1–70 mL), and large infarct volume (>70 mL). Both univariate and multivariable logistic regression analyses were conducted for each subgroup. In addition, we conducted univariate and multivariate logistic regression model analyses on subtypes of HT (HI, PH and sHT). Based on the independent predictors of HT in all patients, a nomogram was constructed using the rms package in R software. Statistical significance was set at a two-tailed *p*-value of ≤0.05. All statistical analyses were performed using R version 4.1.3 and IBM SPSS Statistics 27.

## Results

**Figure 1:**
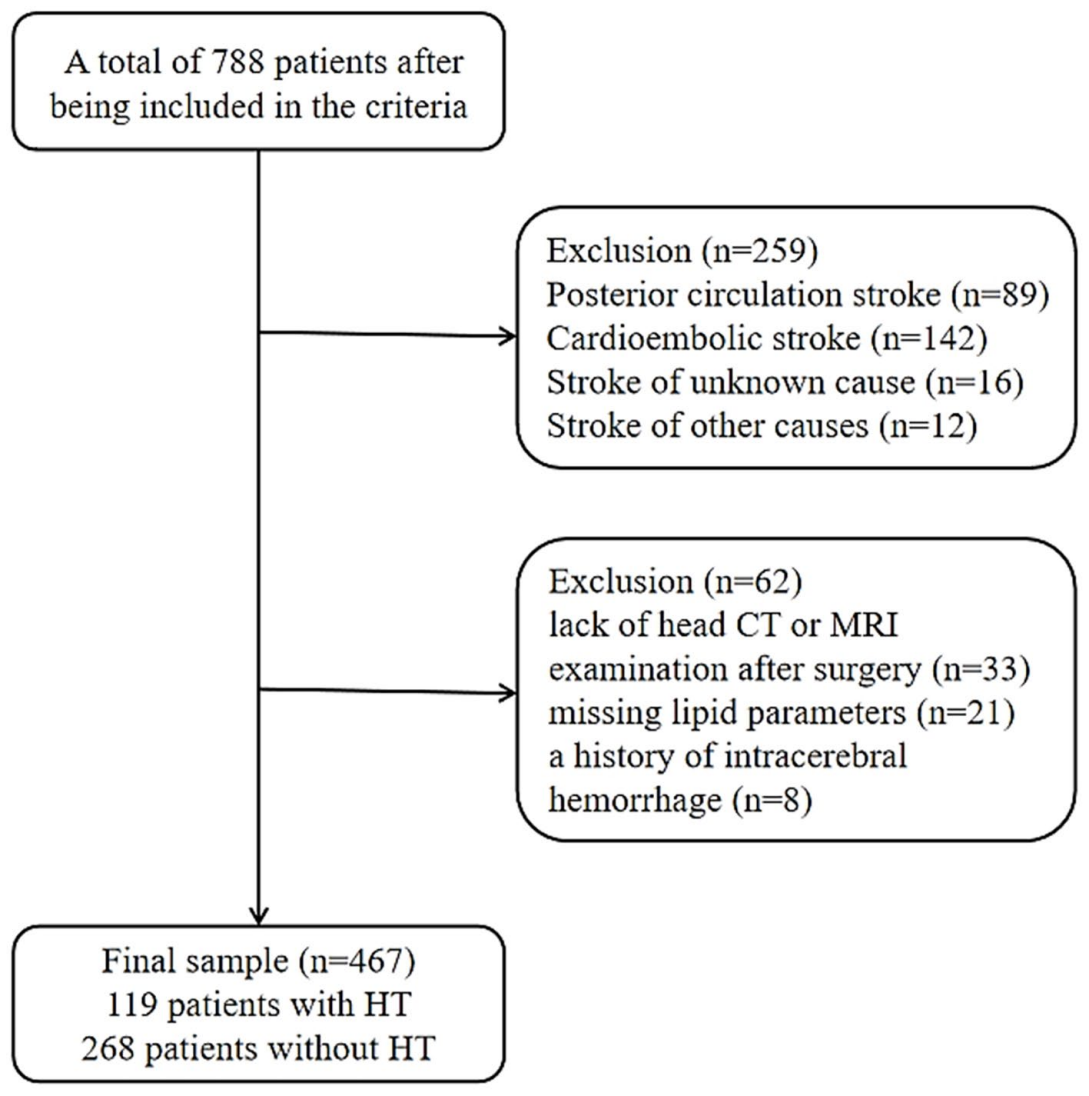
Flow chart of participants’ selection.

A total of 788 patients were screened according to the inclusion criteria. After excluding patients who did not meet the criteria, 467 patients were included in the statistical analysis (Figure 1). The exclusion reasons were as follows: (1) posterior circulation stroke (*n* = 89); (2) cardioembolic (*n* = 142), undetermined (*n* = 16), and other types of stroke (*n* = 12); (3) absence of post-surgery head CT or MRI examination (*n* = 33); (4) missing lipid profile data (*n* = 21); (5) previous history of intracranial hemorrhage (*n* = 8). Among the included patients, 199 (42.6%) experienced HT.

### Baseline characteristics

Table 1 presents a comparison of baseline characteristics between patients with and without HT. Compared to patients who did not experience HT after EVT, those with HT had higher age, NIHSS score, ASPECTS score, systolic blood pressure, number of EVT treatments, infarct volume, and surgery duration, while their AIP, homocysteine, and albumin levels were lower.

**Table 1:** Baseline characteristics according to the presence of HT.

	All patients (*n* = 467)	Without HT (*n* = 268)	With HT (*n* = 199)	*p*-value
Demographic characteristics				
Age mean ± SD, years	63.52 ± 11.56	62.19 ± 11.98	65.32 ± 11.74	0.005
Male *n* (%)	339 (72.6)	198 (73.6)	142 (71.2)	0.606
BMI mean ± SD, Kg/m^2^	24.84 ± 4.02	25.03 ± 3.61	24.57 ± 4.51	0.218
Clinical characteristics				
Smoking *n* (%)	229 (49.0)	136 (50.5)	102 (51.5)	0.767
SBP mean ± SD, mmHg	140.19 ± 20.52	138.32 ± 20.01	142.71 ± 20.97	0.004
DBP mean ± SD, mmHg	82.56 ± 12.96	82.25 ± 13.44	82.98 ± 12.31	0.546
NIHSS median (IQR)	10 (6–16)	8 (5–14)	12 (8–18)	<0.001
ASPECTS median (IQR)	5 (3–6)	5 (4–6)	4 (3–5)	<0.001
Antiplatelet aggregation (IQR)	135 (28.9)	72 (26.9)	63 (31.7)	0.259
Lipid-lowering (IQR)	138 (29.6)	75 (28.0)	63 (31.7)	0.390
Intravenous thrombolysis *n* (%)	120 (25.7)	62 (23.0)	58 (29.2)	0.142
Medical history				
Hypertension *n* (%)	293 (62.7)	163 (60.5)	130 (65.6)	0.234
Diabetes *n* (%)	135 (28.9)	72 (26.7)	63 (31.8)	0.215
History of Stroke *n* (%)	106 (22.7)	67 (24.9)	39 (19.6)	0.168
Coronary heart disease *n* (%)	60 (12.9)	39 (14.4)	21 (10.6)	0.202
Laboratory characteristics				
TC mean ± SD, mmol/L	4.20 ± 0.94	4.25 ± 0.94	4.15 ± 0.94	0.260
TG mean ± SD, mmol/L	1.43 ± 0.66	1.46 ± 0.63	1.37 ± 0.70	0.178
LDL-C mean ± SD, mmol/L	2.67 ± 0.71	2.72 ± 0.72	2.61 ± 0.69	0.093
HDL-C mean ± SD, mmol/L	1.07 ± 0.23	1.05 ± 0.23	1.08 ± 0.24	0.125
AIP mean ± SD	0.10 ± 0.23	0.12 ± 0.23	0.07 ± 0.23	0.026
HbA1c mean ± SD, %	6.56 ± 1.61	6.43 ± 1.51	6.72 ± 1.73	0.054
Homocysteine mean ± SD, μmol/L	22.59 ± 21.59	24.29 ± 25.34	20.29 ± 14.90	0.048
Albumin mean ± SD, g/L	39.49 ± 4.63	39.92 ± 4.57	38.91 ± 4.65	0.019
Surgical characteristics				
OPT median (IQR), min	540 (350–750)	550 (333–728)	520 (380–760)	0.596
Surgery duration median (IQR), min	110 (80–140)	110 (80–134)	120 (80–150)	0.02
The number of endovascular treatments median (IQR)	2 (1–3)	2 (1–3)	3 (2–4)	<0.001
Imaging characteristics				
Infarct volume median (IQR), ml	30.8 (8.9–89.5)	15 (6–46)	72 (20–157)	<0.001

HT, hemorrhagic transformation; IQR, interquartile range; SD, standard deviation; BMI, body mass index; SBP, systolic blood pressure; DBP, diastolic blood pressure; NIHSS, National Institutes of Health Stroke Scale; ASPECTS, Alberta Stroke Program Early CT Score; TG, triglycerides; TC, total cholesterol; LDL-C, low-density lipoprotein cholesterol; HDL-C, high-density lipoprotein cholesterol; AIP, atherogenic index of plasma; HbA1c, hemoglobin A1c; OPT: onset to puncture time.

Supplementary Table S1 displays the incidence rates of HT, HI, and PH within different infarct volume subgroups. The results showed that as infarct volume increased, the incidence of HT, HI, and PH also increased, with HT (*p* < 0.001) and PH (*p* < 0.001) showing the most significant upward trends.

Supplementary Table S2 presents the baseline characteristics of patients with and without HT, categorized by different infarct volume subgroups. Supplementary Table S3 shows the baseline characteristics of patients with both HT subtypes (PH, HI and sHT) compared to those without HT.

### Infarct volume distribution

Supplementary Figure S1 illustrates the distribution histogram of infarct volumes grouped in 15 mL intervals. The majority of infarct volumes were concentrated in the lower range, with a sharp decrease in case frequency as the infarct volume increased. This distribution trend is similar to previous studies (18).

### Logistic regression analysis of potential prognostic factors for HT

Table 2 presents the results of univariate and multivariate logistic regression analyses of potential prognostic factors for HT. The study found that age, NIHSS score, ASPECTS score, systolic blood pressure, number of EVT treatments, infarct volume, surgery duration, AIP, and albumin were significantly associated with HT (*p* < 0.05). Multivariate logistic regression analysis revealed that AIP (OR = 0.20, 95% CI = 0.07–0.53, *p* = 0.001), ASPECTS score (OR = 0.78, 95% CI = 0.67–0.92, *p* = 0.003), infarct volume (OR = 1.00, 95% CI = 1.00–1.01, *p* = 0.036), and number of EVT treatments (OR = 1.52, 95% CI = 1.23–1.87, *p* < 0.001) were significantly associated with HT.

**Table 2:** Univariate and multivariate analyses for the potential prognostic factors according to the presence of HT.

	Univariate analysis	Multivariate analysis	
	*P*-value	OR (95%CI)	*P*-value
Demographic characteristics			
Age (years)	0.004	1.02 (1.00–1.04)	0.096
Male	0.606	-	-
BMI (Kg/m^2)	0.221	-	-
Clinical characteristics			
Smoking	0.767	-	-
SBP (mmHg)	0.023	1.01 (1.00–1.02)	0.103
DBP (mmHg)	0.545	-	-
NIHSS	<0.001	1.03 (0.99–1.08)	0.130
ASPECTS	<0.001	0.78 (0.67–0.92)	0.003
Antiplatelet aggregation	0.259	-	-
Lipid-lowering	0.390	-	-
Intravenous thrombolysis	0.142	-	-
Medical history			
Hypertension	0.235	-	-
Diabetes	0.182	-	-
History of stroke	0.169	-	-
Coronary heart disease	0.203	-	-
Laboratory characteristics			
TC (mmol/L)	0.259	-	-
TG (mmol/L)	0.179	-	-
LDL-C (mmol/L)	0.094	-	-
HDL-C (mmol/L)	0.125	-	-
AIP	0.027	0.20 (0.07–0.53)	0.001
HbA1c (%)	0.056	-	-
Homocysteine (μmol/L)	0.055	-	-
Albumin (g/L)	0.022	0.97 (0.92–1.01)	0.164
Surgical characteristics			
OPT (min)	0.556	-	-
Surgery duration (min)	0.003	1.00 (1.00–1.01)	0.167
The number of endovascular treatments	<0.001	1.52 (1.23–1.87)	<0.001
Imaging characteristics			
Infarct volume (ml)	<0.001	1.00 (1.00–1.01)	0.036

### Subgroup analysis of infarct volume

Table 3 presents univariate and multivariate logistic regression analyses for potential prognostic factors related to HT based on infarct volume subgroups. It was found that only the small infarct volume subgroup (0–15 mL) showed an independent association between AIP (OR = 0.11, 95% CI = 0.02–0.61, *p* = 0.012) and HT after adjusting for other variables. In contrast, AIP did not show a significant independent association with HT in the other infarct volume subgroups (*p* > 0.05).

**Table 3:** Univariate and multivariate analyses for the potential prognostic factors according to the presence of HT categorized by infarct volume groups.

	Small infarct volume group (*n* = 170)			Medium infarct volume group (*n* = 149)			Large infarct volume group (*n* = 148)		
	Univariate analysis	Multivariate analysis		Univariate analysis	Multivariate analysis		Univariate analysis	Multivariate analysis	
	*P*-value	OR (95%CI)	*P*-value	*P*-value	OR (95%CI)	*P*-value	*P*-value	OR (95%CI)	*P*-value
Demographic characteristics									
Age (years)	0.049	1.03 (0.99–1.07)	0.186	0.048	1.01 (0.98–1.05)	0.381	0.162	-	-
Male	0.745	-	-	0.383	-	-	0.239	-	-
BMI (Kg/m^2^)	0.931	-	-	0.003	0.90 (0.81–0.99)	0.034	0.484	-	-
Clinical characteristics									
Smoking	0.291	-	-	0.383	-	-	0.483	-	-
SBP (mmHg)	0.152	-	-	0.238	-	-	0.111	-	-
DBP (mmHg)	0.750	-	-	0.398	-	-	0.476	-	-
NIHSS	0.044	1.06 (0.97–1.15)	0.212	0.030	1.04 (0.97–1.11)	0.292	0.720	-	-
ASPECTS	0.013	0.76 (0.53–1.07)	0.115	0.109	-	-	0.334	-	-
Antiplatelet aggregation	0.623	-	-	0.167	-	-	0.927	-	-
Lipid-lowering	0.719	-	-	0.471	-	-	0.398	-	-
Intravenous thrombolysis	0.757	-	-	0.279	-	-	0.557	-	-
Medical history									
Hypertension	0.495	-	-	0.806	-	-	0.679	-	-
Diabetes	0.924	-	-	0.471	-	-	0.223	-	-
History of stroke	0.551	-	-	0.595	-	-	0.683	-	-
Coronary heart disease	0.665	-	-	0.082	-	-	0.360	-	-
Laboratory characteristics									
TC (mmol/L)	0.369	-	-	0.730	-	-	0.347	-	-
TG (mmol/L)	0.195	-	-	0.189	-	-	0.833	-	-
LDL-C (mmol/L)	0.096	-	-	0.423	-	-	0.367	-	-
HDL-C (mmol/L)	0.114	-	-	0.369	-	-	0.967	-	-
AIP	0.016	0.11 (0.02–0.61)	0.012	0.078	-	-	0.660	-	-
HbA1c (%)	0.713	-	-	0.073	-	-	0.406	-	-
Homocysteine (μmol/L)	0.518	-	-	0.027	0.98 (0.96–1.01)	0.114	0.567	-	-
Albumin (g/L)	0.728	-	-	0.013	0.96 (0.87–1.05)	0.372	0.608	-	-
Surgical characteristics									
OPT (min)	0.234	-	-	0.179	-	-	0.293	-	-
Surgery duration (min)	0.118	-	-	0.108	-	-	0.168	-	-
The number of endovascular treatments	0.007	1.97 (1.28–3.03)	0.002	0.009	1.35 (0.98–1.85)	0.063	0.002	1.64 (1.20–2.25)	0.002

### Analysis of HT subtypes

Table 4 presents the results of univariate and multivariate logistic regression analyses of potential prognostic factors for the HT subtypes (HI, PH and sHT). The analysis revealed that AIP was independently associated with the risk of HI and sHT (OR = 0.12, 95% CI = 0.03–0.47, *p* = 0.003 for HI; OR = 0.11, 95% CI = 0.03–0.46, *p* = 0.002 for sHT), whereas AIP was not independently associated with the risk of PH.

**Table 4:** Univariate and multivariate analyses for the potential prognostic factors according to the presence of HI, PH, and sHT.

	Hemorrhagic infarction (*n* = 72)			Parenchymal hematoma (*n* = 126)			symptomatic hemorrhagic transformation (*n* = 87)		
	Univariate analysis	Multivariate analysis		Univariate analysis	Multivariate analysis		Univariate analysis	Multivariate analysis	
	*P*-value	OR (95%CI)	*P*-value	*P*-value	OR (95%CI)	*P*-value	P-value	OR (95%CI)	*P*-value
Demographic characteristics									
Age mean (years)	0.005	1.03 (1.00–1.06)	0.046	0.052	-	-	0.011	1.02 (0.99–1.04)	0.235
Male	0.396	-	-	0.953	-	-	0.411	-	-
BMI (Kg/m^2^)	0.837	-	-	0.036	0.90 (0.84–0.97)	0.008	0.348	-	-
Clinical characteristics									
Smoking *n*	0.799	-	-	0.767	-	-	0.878	-	-
SBP (mmHg)	0.051	-	-	0.060	-	-	0.042	1.01 (1.00–1.03)	0.058
DBP (mmHg)	0.592	-	-	0.693	-	-	0.724	-	-
NIHSS	<0.001	1.00 (0.95–1.06)	0.892	<0.001	1.05 (1.00–1.10)	0.072	<0.001	1.02 (0.97–1.08)	0.446
ASPECTS	<0.001	0.76 (0.61–0.95)	0.018	<0.001	0.80 (0.65–0.97)	0.024	<0.001	0.84 (0.67–1.03)	0.120
Antiplatelet aggregation	0.191	-	-	0.527	-	-	0.452	-	-
Lipid-lowering	0.119	-	-	0.941	-	-	0.943	-	-
Intravenous thrombolysis	0.221	-	-	0.007	0.59 (0.34–1.01)	0.056	0.848	-	-
Medical history									
Hypertension	0.011	0.54 (0.28–1.05)	0.069	0.909	-	-	0.645	-	-
Diabetes	0.127	-	-	0.383	-	-	0.642	-	-
History of stroke	0.431	-	-	0.180	-	-	0.550	-	-
Coronary heart disease	0.275	-	-	0.338	-	-	0.929	-	-
Laboratory characteristics									
TC (mmol/L)	0.089	-	-	0.706	-	-	0.055	-	-
TG (mmol/L)	0.141	-	-	0.379	-	-	0.063	-	-
LDL-C (mmol/L)	0.043	0.75 (0.47–1.20)	0.233	0.393	-	-	0.022	0.68 (0.45–1.03)	0.069
HDL-C (mmol/L)	0.947	-	-	0.045	3.40 (1.15–10.06)	0.027	0.317	-	-
AIP	0.038	0.12 (0.03–0.47)	0.003	0.105	-	-	0.031	0.11 (0.03–0.46)	0.002
HbA1c	0.296	-	-	0.265	-	-	0.194	-	-
Homocysteine (μmol/L)	0.249	-	-	0.081	-	-	0.171	-	-
Albumin (g/L)	0.032	1.01 (0.93–1.08)	0.899	0.019	0.95 (0.90–1.01)	0.098	0.035	0.96 (0.90–1.03)	0.259
Surgical characteristics									
OPT (min)	0.233	-	-	0.909	-	-	0.971	-	-
Surgery duration median (min)	0. 013	1.01 (1.00–1.02)	0.118	0.019	1.00 (1.00–1.01)	0.392	0.201	-	-
The number of endovascular treatments	<0.001	1.93 (1.42–2.63)	<0.001	<0.001	1.46 (1.16–1.85)	0.002	<0.001	1.80 (1.39–2.33)	<0.001
Imaging characteristics									
Infarct volume (ml)	<0.001	1.00 (1.00–1.01)	0.252	<0.001	1.00 (1.00–1.01)	0.042	<0.001	1.01 (1.00–1.01)	0.004

### The establishment of a nomogram for predicting HT

**Figure 2:**
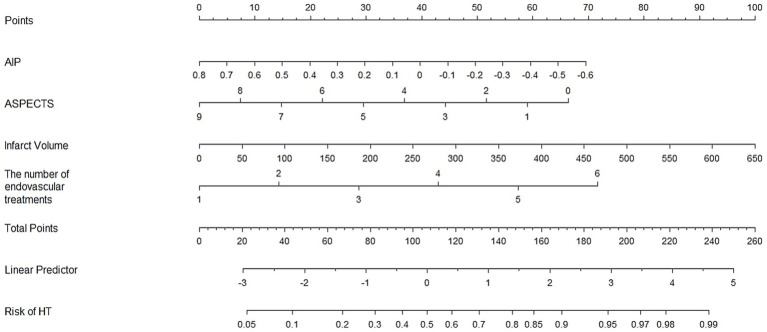
Establishment of a nomogram to predict the incidence of HT.

Based on the analysis of all patients, the factors that remained statistically significant after adjustment for multivariable logistic regression include AIP, ASPECTS, infarct volume, and the number of endovascular treatments. Using these factors, we developed a nomogram to predict the incidence of HT, as shown in Figure 2.

## Discussion

This study found that AIP levels are associated with an increased risk of HT after EVT in patients with large artery atherosclerotic stroke. Additionally, in subgroup analysis, a significant association between lower AIP levels and HT was observed in the small infarct volume group (≤15 mL), while this association weakened in the medium infarct volume group (15.1–70 mL) and the large infarct volume group (>70 mL). In another subgroup analysis, lower AIP levels were associated with the HI and sHT subtype of HT, but not with the PH subtype.

The exact mechanisms by which lipid parameters increase HT risk remain unclear, but several mechanisms could be involved. Lipids are important components of the vascular wall and blood–brain barrier (BBB) and may play a role in maintaining the integrity of cerebral blood vessels and the BBB. Studies have shown that low serum lipid levels can lead to increased erythrocyte permeability and vascular leakage (19, 20). Other studies have suggested (21) that high triglyceride levels contribute to increased blood viscosity and promote the generation of coagulation factors VII and IX, as well as plasminogen activator inhibitor (PAI). Furthermore, HDL-C is known to activate the fibrinolytic system, reduce platelet activation, and inhibit thromboxane A2 synthesis, thereby reducing platelet aggregation (22). These factors may all contribute to an increased risk of HT.

However, the mechanisms behind HT are highly complex, especially in patients with larger infarct volumes. Large infarcts involve more extensive vascular and brain parenchymal damage, as well as more complex pathological changes, including reoxidative stress, and inflammatory cascades. Oxidative stress and inflammatory cascades induce neuroinflammatory responses and activate multiple cell death pathways, further promoting BBB disruption and increased vascular permeability (23–25). matrix metalloproteinases (MMPs) are enzymes that degrade extracellular matrices, primarily activated during the inflammatory response following ischemia. These proteases can degrade the vascular basement membrane and extracellular matrices, leading to vascular wall fragility and rupture, thus increasing the risk of bleeding (26). These factors often play a dominant role in the onset of HT. For patients with smaller infarct volumes, the brain damage is relatively mild, and the pathological changes are fewer, so the occurrence of hemorrhage is more likely to be influenced by lipid metabolism. Moreover, our study found that PH subtype of HT is more common in patients with larger infarct volumes, suggesting that the underlying mechanisms of HI subtype of HT may be similar to those described above.

As a comprehensive lipid metabolism index, AIP considers the ratio of TG to HDL-C, reflecting the dynamic balance of lipids. This makes AIP a more comprehensive marker of lipid metabolism, potentially better revealing the role of lipid imbalance in atherosclerosis (27). In this study, a lower AIP level was found to be significantly associated with HT.

Previous studies have explored the role of lipid parameters in post-EVT HT. For instance, a study by Jingping Sun et al. involving 384 patients found (28) that low TG levels were associated with brain PH post-surgery. Another study by Jie Li et al. involving 155 patients suggested (29) that a combination of lower TG and higher HDL-C levels could predict PH after thrombectomy. However, in this study, only HDL-C levels among traditional lipid parameters were associated with PH in HT. Additionally, some researchers have observed an independent association between LDL-C levels ≤50 mg/dL and delayed post-thrombectomy PH, but LDL-C did not predict immediate post-thrombectomy HT (30, 31).

The incidence of HT in this study was 42.6%, which is relatively high compared to previous studies reporting 49.3 and 47.9% after EVT (4, 32). The median infarct volume in this study was 30.8 mL, compared to a median of 24.9 mL in previous studies involving EVT (33). It is possible that hospitals tend to prioritize emergency surgery for patients with larger infarct volumes and more severe conditions, which may explain the higher severity of cases in this study. The occurrence of HT could also be influenced by the techniques and skill levels of different operators. Moreover, in addition to the 24-h post-surgery imaging examination, patients undergo follow-up imaging at 7 days post-surgery, which may also affect the statistical incidence of HT.

This study did not specifically analyze the impact of AIP on short- or long-term prognosis, particularly in patients who have experienced HT. However, since previous studies have shown that PH is associated with clinical prognosis while HI is generally benign and does not significantly affect prognosis (4, 34), it can be inferred that low AIP levels may not increase the risk of poor prognosis.

Although this study provides strong evidence for the association between AIP and HT, there are several limitations. Firstly, the manual segmentation method for measuring infarct volume may introduce human error, affecting the precision of the volume calculation. Furthermore, CT’s spatial resolution is insufficient for accurately measuring small infarct regions, which may impact the accuracy of infarct volume estimation.

In addition to imaging limitations, there are other study limitations. First, certain related factors, such as the presence of core infarction before treatment and postoperative blood pressure fluctuations, were not included in the analysis. These factors may affect HT. Second, we are unable to establish a causal relationship between AIP and HT, and the dynamic changes in AIP values during the early stages of ischemic stroke were not considered, nor was their potential effect on post-surgical HT risk. Lastly, the study’s regional and demographic limitations may affect the external validity and generalizability of the results.

## Conclusion

This study indicates that AIP is significantly associated with the risk of HT in patients with LAA after EVT. The findings suggest that AIP can serve as an important biomarker for predicting HT risk, particularly in patients with smaller infarct volumes (≤15 mL). Moreover, AIP is closely related to the HI and sHT subtype of HT.

## Data Availability

The original contributions presented in the study are included in the article/Supplementary material, further inquiries can be directed to the corresponding author/s.
