# Supplementary material for: The impact of low plasma atherosclerosis index on hemorrhagic transformation after endovascular treatment of large artery atherosclerotic stroke
Source: Front Neurol. 2025 Nov 6;16:1694640. doi: 10.3389/fneur.2025.1694640 (PMC12635849; doi:10.3389/fneur.2025.1694640)
Supplement: Supplementary file 2 [file Data_Sheet_1.pdf]

Table S1 Clinical outcomes in each infarct volume groups

	Small Infarct Volume Group (0-15 ml), n=170	Medium Infarct Volume Group (15.1-70 ml), n=149	Large Infarct Volume Group (>70 ml), n=148	p-value
HT (%)	37 (21.8%)	62 (41.6%)	100 (67.6%)	<0.001
HI (%)	18 (10.6%)	28 (18.8%)	26 (17.6%)	0.088
PH (%)	19 (11.2%)	34 (22.8%)	74 (50.0%)	<0.001

Abbreviations: HT, Hemorrhagic Transformation; HI, Hemorrhagic Infarction; PH, Parenchymal Hematoma

Table S2 Baseline Characteristics according to the presence of HT with different Infarct Volume Groups

	Small Infarct Volume Group (n=170)			Medium Infarct Volume Group (n=149)			Large Infarct Volume Group (n=148)		
	Without HT (n=133)	HT (n=37)	P-value	Without HT (n=87)	HT (n=62)	P-value	Without HT (n=48)	HT (n=100)	P-value
Demographic characteristics									
Age mean ± SD, years	62.86±11.15	66.84±8.25	0.020	61.38 ±14.20	65.6±9.80	0.034	61.79 ±9.74	64.60 ±12.06	0.238
Male n (%)	104 (78.1)	28 (76.7)	0.745	62 (71.2)	40 (64.5)	0.382	31 (64.5)	74 (74.0)	0.162
BMI mean ± SD, Kg/m^2	24.71±3.30	23.93±3.46	0.211	25.59±4.31	23.47± 3.65	0.002	24.92 ±2.94	25.49 ±5.14	0.399
Clinical characteristics									
Smoking n (%)	66 (49.6)	22 (59.4)	0.290	47 (54.0)	29 (46.7)	0.383	22 (45.8)	52 (52.0)	0.482
SBP mean ± SD, mmHg	139.50 ±19.09	144.76 ±21.23	0.150	137.51± 21.10	141.44± 18.31	0.239	136..4 ±20.69	142.74 ±22.50	0.110
DBP mean ± SD, mmHg	84.07± 13.11	84.86±14.89	0.751	78.85±13.59	81.55±9.61	0.373	81.54 ±13.61	83.17 ±12.77	0.478
NIHSS median (IQR)	6 (4-9)	8 (5-10)	0.049	8 (6-14)	12 (8-16)	0.005	16 (12-19)	16 (10-20)	0.666
ASPECTS median (IQR)	6 (5-7)	5 (5-6)	0.012	5 (3-5)	4 (3-5)	0.170	3 (3-4)	3 (2-4)	0.149
Antiplatelet Aggregation (IQR)	34 (25.6)	8 (21.6)	0.623	23 (26.4)	23 (37.1)	0.165	15 (31.3)	32 (32.0)	0.927
Lipid-lowering (IQR)	40 (30.1)	10 (27.0)	0.719	22 (25.3)	19 (30.6)	0.470	13 (27.1)	34 (34.0)	0.397
Intravenous Thrombolysis n (%)	32 (24.0)	8 (21.6)	0.757	16 (18.3)	16 (25.8)	0.277	14 (29.1)	34 (34.0)	0.557
Medical History									
Hypertension n (%)	78 (58.6)	24 (64.8)	0.495	53 (60.9)	39 (62.9)	0.806	31 (64.5)	68 (68.0)	0.679
Diabetes n (%)	37 (27.8)	10 (27.0)	0.924	22 (25.2)	19 (30.6)	0.470	12 (25.0)	35 (35.0)	0.221
History of Stroke n (%)	39 (29.3)	9 (24.3)	0.550	17 (19.5)	10 (16.1)	0.594	11 (22.9)	20 (20.0)	0.683
Coronary Heart Disease n (%)	14 (10.5)	3 (8.81)	0.665	16 (18.3)	5 (8.0)	0.074	9 (18.7)	13(13.0)	0.357
Laboratory Characteristics									
TC mean ± SD, mmol/L	4.22±0.95	4.06± 0.85	0.371	4.25± 0.86	4.20±0.78	0.732	4.32 ±1.07	4.14 ±1.05	0.348
TG mean ± SD, mmol/L	1.44±0.31	1.28±0.83	0.194	1.50±0.70	1.35±0.65	0.188	1.46 ±0.55	1.43 ±0.68	0.834
LDL-C mean ± SD, mmol/L	2.70±0.72	2.49±0.59	0.095	2.72±0.71	2.64±0.57	0.426	2.76 ±0.73	2.64 ±0.79	0.369
HDL-C mean ± SD, mmol/L	1.03± 1.24	1.10±0.21	0.111	1.08±0.20	1.11±0.22	0.371	1.06 ±0.24	1.06 ±0.25	0.968
AIP mean ± SD	0.12±0.25	0.00±0.27	0.014	0.11±0.21	0.05±0.20	0.075	0.12 ±0.19	0.11 ±0.22	0.662
HbA1c mean ± SD, %	6.49± 1.62	6.38±1.63	0.714	6.24±1.30	6.72±1.83	0.084	6.61±1.53	6.85±1.70	0.407
Homocysteine mean ± SD, μmol/L	23.81± 27.25	20.77±13.88	0.514	27.64±27.46	18.63±10.55	0.006	19.56 ±11.74	21.15±17.41	0.568
Albumin mean ± SD, g/L	39.89±4.03	40.14±3.73	0.730	40.34±5.66	38.30±3.01	0.005	39.29±3.71	38.83±5.64	0.609
Surgical Characteristics									
OPT median (IQR), min	530 (347.5-710)	590 (435-795)	0.114	560 (300-740)	610 (418-780)	0.180	585 (325-833)	475 (310-678)	0.251
surgery duration median (IQR), min	105 (105-130)	120 (90-140)	0.119	110 (90-140)	120 (83-150)	0.249	110 (83-141)	120 (80-150)	0.333
the number of endovascular treatments median (IQR)	2 (1-3)	3 (2-3)	0.011	2 (1-3)	3 (1-4)	0.010	2 (1-3)	3 (2-4)	0.001

Abbreviations: HT, Hemorrhagic Transformation; IQR, interquartile range; SD, standard deviation; BMI, Body Mass Index; SBP, systolic blood pressure; DBP, diastolic blood pressure; NIHSS, National Institutes of Health Stroke Scale; ASPECTS, Alberta Stroke Program Early CT Score; TG, Triglycerides; TC, Total Cholesterol; LDL-C, Low-Density Lipoprotein Cholesterol; HDL-C, High-Density Lipoprotein Cholesterol; AIP, Atherogenic Index of Plasma; HbA1c, Hemoglobin A1c; OPT: Onset to Puncture Time

Table S3 Baseline Characteristics according to the presence of HI, PH and sHT

	Without HT (n=268)	With HI (n=72)	P-value	With PH (n=127)	P-value	With sHT (n=87)	P-value
Demographic characteristics							
Age mean ± SD, years	62.19±11.98	66.64±10.60	0.004	64.64±10.79	0.051	66.00±11.62	0.010
Male n (%)	198 (73.6)	49 (68.0)	0.395	93 (73.2)	0.953	60 (69.0)	0.410
BMI mean ± SD, Kg/m^2	25.03±3.61	525.15±5.76	0.837	24.21±3.57	0.035	24.55±5.62	0.349

Clinical characteristics							
Smoking n (%)	136 (50.5)	37 (51.3)	0.799	66 (52.0)	0.767	43 (49.4)	0.878
SBP mean ± SD, mmHg	138.32±20.01	143.64±22.21	0.050	142.43±20.42	0.059	143.34±19.45	0.041
DBP mean ± SD, mmHg	82.25±13.44	83.19±13.20	0.593	82.80±11.79	0.694	82.80±10.70	0.693
NIHSS median (IQR)	8 (5-14)	11 (7-17)	<0.001	13 (9-19)	<0.001	14 (9-18)	<0.001
ASPECTS median (IQR)	4 (3-5)	4 (3-5)	<0.001	4 (3-5)	<0.001	4 (3-5)	<0.001
Antiplatelet Aggregation (IQR)	72 (26.9)	25 (34.7)	0.190	38 (29.9)	0.527	27 (31.0)	0.451
Lipid-lowering (IQR)	75 (28.0)	27 (37.5)	0.118	36 (28.6)	0.941	24 (27.6)	0.943
Intravenous Thrombolysis n (%)	62 (23.0)	12 (16.6)	0.219	46 (36.2)	0.006	21 (24.1)	0.848
Medical History							
Hypertension n (%)	163 (60.5)	55 (76.3)	0.010	76 (60.0)	0.909	55 (63.2)	0.645
Diabetes n (%)	72 (26.7)	25 (34.7)	0.144	39 (30.7)	0.383	25 (28.7)	0.682
History of Stroke n (%)	67 (24.9)	15 (20.8)	0.430	24 (19.0)	0.179	19 (21.8)	0.550
Coronary Heart Disease n (%)	39 (14.4)	7 (9.7)	0.271	14 (11.0)	0.337	13 (14.9)	0.929
Laboratory Characteristics							
TC mean ± SD, mmol/L	4.25±0.94	1.04±0.80	0.089	4.21±1.00	0.706	4.02±1.00	0.054
TG mean ± SD, mmol/L	1.46±0.63	1.33±0.80	0.139	1.40±0.64	0.380	1.31±0.68	0.062
LDL-C mean ± SD, mmol/L	2.72±0.72	2.53±0.61	0.042	2.66±0.73	0.394	2.51±0.74	0.021
HDL-C mean ± SD, mmol/L	1.05±0.23	1.05±0.21	0.947	1.10±0.25	0.044	1.08±0.27	0.074
AIP mean ± SD	0.12±0.23	0.05±0.25	0.037	0.77±0.21	0.104	0.06±0.23	0.030
HbA1c mean ± SD, %	6.43±1.51	6.90±1.94	0.061	6.62±1.59	0.265	6.68±1.67	0.192
Homocysteine mean ± SD, μmol/L	24.29±25.34	20.64±17.14	0.247	20.04±13.47	0.030	20.30±15.80	0.168
Albumin mean ± SD, g/L	39.92±4.57	39.31±4.04	0.297	38.69±4.96	0.015	38.65±5.28	0.031
Surgical Characteristics							
OPT median (IQR), min	550 (333-728)	570 (445-770)	0.115	490 (330-750)	0.611	500 (330-720)	0.776
surgery duration median (IQR), min	110 (80-134)	120 (90-145)	0.015	120 (80-150)	0.161	110 (80-150)	0.547
the number of endovascular treatments median (IQR)	2 (1-3)	3 (2-4)	<0.001	3 (2-4)	<0.001	3 (2-4)	<0.001
Imaging Characteristics							
Infarct Volume median (IQR), ml	15 (6-46)	49 (15-98)	<0.001	90 (26-166)	<0.001	92 (28-174)	<0.001

Abbreviations: HT, Hemorrhagic Transformation; IQR, interquartile range; SD, standard deviation; BMI, Body Mass Index; SBP, systolic blood pressure; DBP, diastolic blood pressure; NIHSS, National Institutes of Health Stroke Scale; ASPECTS, Alberta Stroke Program Early CT Score; TG, Triglycerides; TC, Total Cholesterol; LDL-C, Low-Density Lipoprotein Cholesterol; HDL-C, High-Density Lipoprotein Cholesterol; AIP, Atherogenic Index of Plasma; HbA1c, Hemoglobin A1c; OPT:Onest to Puncture Time

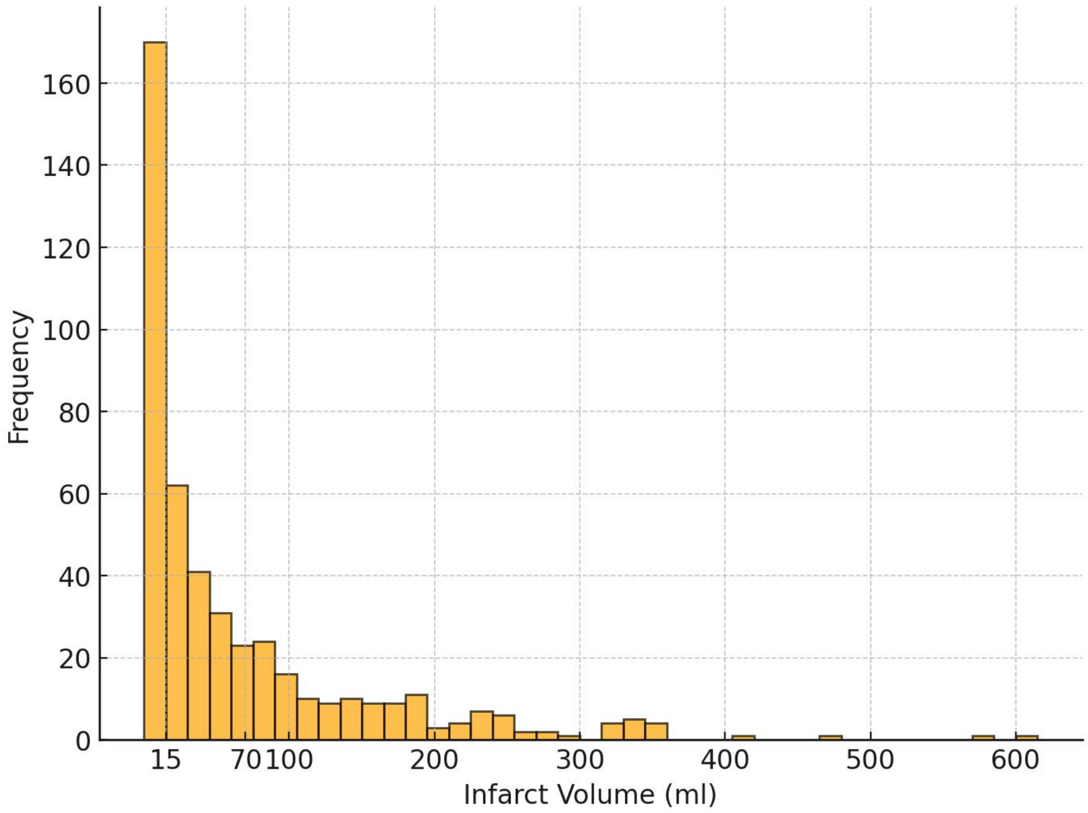

Figure S1 Distribution of infarct volumes in the patient sample
